# The antiangiogenic activities of ethanolic crude extracts of four *Salvia* species

**DOI:** 10.1186/1472-6882-13-358

**Published:** 2013-12-13

**Authors:** Malek Zihlif, Fatma Afifi, Rana Abu-Dahab, Amin Malik Shah Abdul Majid, Hamza Somrain, Mohanad M Saleh, Zeyad D Nassar, Randa Naffa

**Affiliations:** 1 Department of Pharmacology, Faculty of Medicine, The University of Jordan, Amman 11942, Jordan; 2 Department of Pharmaceutical Sciences, Faculty of Pharmacy, The University of Jordan Amman, Jordan; 3 Department of Pharmacology, School of Pharmaceutical Sciences, Universiti Sains Malaysia, Minden 11800, Penang, Malaysia; 4 University of Queensland, School of Pharmacy, 20 Cornwall Street, Woolloongabba, QLD 4102, Australia; 5 Department of Physiology and Biochemistry, Faculty of Medicine, The University of Jordan, Amman 11942, Jordan

**Keywords:** *Salvia triloba*, *Salvia hormium*, *Salvia dominica*, *Salvia syriaca*, Antiangiogenesis, MCF 7, Jordan

## Abstract

**Background:**

Angiogenesis is one of cancer hallmarks that are required for both cancer progression and metastasis. In this study we examined the antiangiogenic properties of the ethanolic crude extracts of four *Salvia* species grown in Jordan.

**Methods:**

The direct antiangiogenic activity was evaluated using various models: *ex vivo* rat aortic ring assay, *in vitro* assessment of HUVEC proliferation and migration, and in *vivo* CAM assay, while we used the changes in the expression of HIF-1α and VEGF in breast cancer cells (MCF 7) as an indicative for the indirect antiangiogenic activity.

**Results:**

All four crude extracts showed a potential antiangiogenic activity in the rat aortic assay, however two species were found to be cytotoxic against Fibroblast cell line (PLF); the finding that caused the exclusion of these two extracts from further studies. Of the two remaining extracts, *S. triloba* showed very promising direct and indirect antiangiogenic activities. *S. triloba* inhibited the HUVEC proliferation with an IC_50_ of 90 μg/mL and HUVEC migration by 82% at 150 μg/mL. Furthermore, the *in vivo* CAM assay also illustrated the high impact of *S. triloba* against the newly formed vessel in the chicken embryonic membrane. Interestingly, the *S. triloba* inhibited the expression of VEGF at the mRNA and protein and the HIF-1α mRNA in the MCF 7 breast cancer cells under both normoxic and hypoxic conditions.

**Conclusions:**

Taken together, all these findings of the direct and indirect angiogenic investigations nominated *S. triloba* as a highly potent antiangiogenic plant that may have chemotherapeutic and/or chemoprevention potentials.

## Background

Angiogenesis is the formation of new blood vessels. Cancer is angiogenesis dependent; any significant increment in tumour size must be in synchrony with increment in the blood supply. The new blood vessels supply the tumour cells with extra amount of oxygen and nutrients, and most importantly they facilitate cancer cell metastasis to other localities [1]. All solid tumours are dependent on the angiogenesis for tumour growth and metastasise [2]. Breast cancer is the most common cancer in women counting for approximately 27% of all new cancers [3]. It is characterized by a distinct metastatic pattern [4]. Cancer cells within the tumour use the newly formed blood vessels as a port to metastasize to other localities [5]. Although less than 0.05% of circulating tumour cells has a potential to become stable metastases [6], the vast majority of breast cancer-related deaths result from metastatic tumours [7]. Angiogenesis, one of cancer hallmarks is involved in the progression and metastasis of breast cancer [8]. The process is tightly controlled through a balance of positive and negative regulatory factors and is triggered by pro-angiogenic growth factors [9]. Vascular endothelial growth factor (VEGF) is a major mediator of angiogenesis in cancer [10]. VEGF expression is induced under hypoxic conditions; a multistage process, in which the alpha subunit of hypoxia inducible factor-1 (HIF-1α) plays an important role [10]. Under normoxic condition, HIF-1α rapidly decreases since it is bound to the tumour suppressor Von Hippel-Lindau (VHL) protein, which in turn results in HIF-1α ubiquitynation and becomes a target for the proteosome. However, the low blood supplies toward the tumour mass drives the tumour tissue into hypoxia, which may induce transcriptional activation of VEGF expression through HIF-1α stabilization [11]. Since angiogenesis plays a prominent role in tumour growth and metastasis, inhibition of angiogenesis is considered as an important strategy for cancer therapy [12]. Furthermore, inhibiting angiogenesis before it starts, which known as angioprevention, has the potential to block the expansion of hyperplastic foci and subsequent tumour development at the premalignant stage [13]. Angioprevention compounds work through diverse pathways, amongst those is the interference with expression of the VEGF and HIF-1α, which stand out among the diverse factors that drive angiogenesis [13].

The genus *Salvia* is the largest and the most important genus of the family Lamiaceae. This genus has about 900 species, widespread throughout the world, and includes several ornamental, culinary and medicinal species [14]. Nineteen species of *Salvia* are reported to occur in the flora of Jordan [15]. Published data indicated that many *Salvia* species exert diverse biological activities, and have been used in many parts of the world as part of the local traditional medicines to treat various conditions. Examples of pharmacological properties are antioxidant [16], antimicrobial [17], anti-inflammatory, analgesic [18], antipyretic, hemostatic [19], hypoglycemic [20] and anti-tumour [21] activities. *S. miltiorrhiza* Bunge, commonly used in traditional Chinese herbal medicine to treat cardiovascular and hepatic disorders, has been shown recently to have significant anti-tumour and anti-angiogenesis properties [22]. Interestingly, the same plant, *S. miltiorrhiza,* has demonstrated some pro-antiangiogenic activity*.* Another recent publication has also reported antiangiogenic activity for *S. officinalis*[23].

Encouraged from the promising results of *S. officinalis,* the current study was designed to evaluate the antiangiogenic activity of four *Salvia* species grown in Jordan. Ethanolic crude extracts of the leaves of *S. dominica, S. syriaca, S. triloba* and *S. hormium* were prepared and their direct antiangiogenic properties were investigated using rat aortic ring assay. Plant extracts that exhibited direct antiangiogenic activities, were further explored for their direct activity in human umbilical endothelial cells (HUVEC) cytotoxicity assay, migration assay and chicken embryo chorioallantoic membrane (CAM) assay. Moreover, to test the extracts’ indirect antiangiogenic activity, the effect on the expression of the VEGF protein and expression of both, VEGF and HIF-1α mRNA was examined using MCF 7 breast cancer cell line. All extracts were phytochemically screened using thin layer chromatography (TLC) and the presence of detected classes of secondary metabolites was reported. The effects of the extracts on normal cells were tested using periodontal fibroblast cell line (PLF).

## Methods

### Plant samples collection and extraction

Plant samples were collected during early flowering period (spring/summer 2009) from different areas in Jordan and have been identified by Prof. Dr. Barakat Abu Iremaileh (Faculty of Agriculture, The University of Jordan). Voucher specimens were deposited at the Department of Pharmaceutical Sciences, Faculty of Pharmacy, The University of Jordan. Plant samples were cleaned from extraneous material and dried at room temperature. Each 2.5 g of coarsely powdered plant material was extracted by refluxing with 25 mL 70% ethanol for 30 min and kept overnight at room temperature before filtration. After filtration, ethanol was evaporated until dryness and the crude extracts were weighed. The crude extracts were dissolved in dimethyl sulphoxide (DMSO) to a final stock concentration of 10 mg/mL. All extracts were kept at 4°C until the time of experiment.

### Cell lines and cell culture

The human breast adenocarcinoma (MCF 7) was purchased from the European Collection of Animal Cell Culture (ECACC). Fibroblast cell line provided kindly from the Faculty of Dentistry, Jordan University of Science and Technology. Human umbilical vein endothelial cell line (HUVEC) was purchased from American Type Cell Culture Collection (ATCC). The MCF 7 and fibroblast cell lines were cultured in DMEM/F12 and DMEM medium (Gibco, Invitrogen, USA), respectively. Media were supplemented with 10% fetal bovine serum (FBS) (Gibco, Invitrogen, USA), 1% of 2 mM L-glutamine, 50 IU/mL penicillin and 50 μg/mL streptomycin (Lonza, Belgium). HUVECs were cultured in F12K medium (Gibco, Invitrogen, USA) supplemented with 0.1 g/mL Heparin (Sigma, USA) and 1% EGFR (Sigma, USA) 10% FBS, 1% of 2 mM L-glutamine, 50 IU/mL penicillin and 50 μg/mL streptomycin. HUVECs from passages 2 through 4 were used through this study. All cells were maintained at 37°C, 5% CO_2_ in a humidified incubator.

### Rat aortic ring assay

The rat aortic ring assay experiment was conducted after the experimental procedures were revised and approved by the Animal Ethics Committee of The University of Jordan. The assay was performed according to the standard protocol of Brown *et al.*[24], with minor modifications. Twelve to fourteen weeks old Sprague Dawley male rats were obtained from the animal house facility of the Faculty of medicine, The University of Jordan (UJ). The animals were humanely sacrificed via cervical dislocation under anesthesia with diethyl ether. Thoracic aorta was excised, rinsed with serum free media, cleaned from the fibroadipose tissue and was cross sectioned into thin rings of 1 mm thickness. M199 basal medium (Gibco, Invitrogen, USA) was used for the lower layer after adding fibrinogen and aprotinin (Sigma, USA) at 3 mg/mL and 5 μg/mL, respectively. A 300 μl of M199 medium was loaded in each 48-well plate and one aortic ring was seeded in each well. To each well, 10 μl of thrombin (Sigma, USA); prepared at 50 NIH U/mL in 0.15 M NaCl: bovine serum albumin (1%); was added and then was allowed to solidify at 37°C in 5% CO_2_ for 60–90 min. The top layer medium was prepared by adding the following to M199 basal medium: 20% of heat inactivated fetal bovine serum (HIFBS) (Gibco, Invitrogen, USA), 1% L-glutamine (Lonza, Belgium), 0.1% aminocaproic acid (Sigma, USA), 1% amphotericin B (Lonza, Belgium) and 0.6% gentamicin (Lonza, Belgium). Plant extracts were added to the top layer medium at concentration of 100 μg/mL. The tissue rings were incubated at 37°C, 5% CO_2_ in a humidified incubator. On day 4, the top layer medium was changed with fresh medium prepared as previously mentioned. The DMSO (1% v/v) and Suramin (100 μg/mL) were used as negative and positive controls respectively. The results examined microscopically at appropriate magnification and the magnitude of blood vessel outgrowth was quantified using Leica Quin software package [25], according to the technique developed by Nicosia *et al.*[26]. The results are presented as mean percent inhibition to the negative control ± SD, (n = 3).

### In vitro cytotoxicity assay

Plant extracts were tested for cytotoxicity against fibroblast cell line. Cells were seeded at density of 10,000 cells/well in 96-well plates. Afterwards, the cells were treated with two concentrations; 50 and 100 μg/mL in quadricate. Control wells contained DMSO at same concentrations. After 72 h incubation, cell viability was determined by MTT assay according to cell proliferation assay kit (Promega, USA). Absorbance (OD) was measured at 570 nm with background subtraction at 630 nm.

### Antiproliferative activity

HUVECs were seeded at a density of 10 × 10^3^ cells/well in 96-well plates and allowed to attach overnight. Plant extracts that showed antiangiogenic activity with aortic ring assay were screened on HUVECs for their IC_50_. Cells were treated with different concentrations (1.5-100 μg/mL) in quadricate. After 72 h treatment, MTT assay was performed according to cell proliferation assay kit (Promega, USA). Absorbance (OD) was measured at 570 nm with background subtraction at 630 nm. The calculation of IC_50_ was performed using a sigmoidal plotting function provided in the GraphPad Prism software. DMSO was used as a negative control.

### Wound healing migration assay

The migration assay was carried out as described previously [27]. Briefly, HUVECs were seeded at 5 × 10^5^/well in 6-well plates and allowed to form a confluent monolayer. The layer of cells was then scraped with a 20–200 μl micropipette tip to create a wound of ~1 mm width. Cells were then washed twice with PBS and replaced with medium containing 100 AND 150 μg/mL of the plant extracts. The wounds were photographed at 0, 12 and 18 h, and the number of migrated cells was counted. Ten readings per well were taken. The results are presented as mean percentage of migration inhibition to the control ± SD, (n = 3).

### *In vivo* CAM assay

Antiangiogenic effect of the plant extracts was investigated *in vivo* using CAM assay as mentioned previously [28]. Five day-old fertilized eggs were obtained from local hatchery. 5 mL of albumin was aspirated and the eggs were incubated horizontally to allow the CAM to detach from the shell. Chosen extracts were prepared in 1.2% agarose discs at concentration of 100 μg/disc. Discs containing the vehicle only (DMSO) were used as negative control. A small window opening was made in the shell, and the discs were directly applied onto the CAM. The square opening was covered with sterilized surgical tape and the embryos were incubated for 48 h at 37°C. The CAMs were photographed under a dissecting microscope and blood vessels in each CAM were counted. The results are presented as a mean percentage of inhibition to the control ± SD, (n = 3).

### Expression of VEGF and HIF-1α

#### MCF 7 cells treatment

MCF 7 cells were seeded at a concentration of 3 × 10^6^ cells in T75 Flask on the day before treatment. Then, the medium was replaced with a new medium, containing three concentrations of plant extract 100, 200 and 300 μg/mL. The cells were incubated at 37°C, 5% CO_2_ for 16 h, under two conditions; hypoxic (0.1% O_2_) and normoxic conditions (20% O_2_). However, before cells were exposed to the hypoxic condition, they were treated under normoxic condition for 1 h then maintained under hypoxic conditions for 16 h. Hypoxic condition was performed by incubating the cells in GasPak Pouch (Becton Dickinson, Sparks, Md., USA). DMSO with the same concentrations as the extracts were then used as negative controls. Then, cells were harvested for RNA extraction.

#### RNA extraction and cDNA synthesis

Total RNA was extracted using Trizol, LS (Invitrogen, USA). The RNA quality was assessed by spectrophotometric method (A_260_/A_280_). RNA samples were stored at -80°C until used. Complementary DNA (cDNA) was synthesized from 1.0 μg total RNA using RT-PCR Kit (Promega, USA) in a final volume of 20 μl using random primers according to the manufacturer's instructions.

#### Quantitative real-time PCR (Q-PCR)

Q-PCR was carried out in IQ4 real time PCR (BioRad, USA). The reaction mixture consisted of 1X GoTaq qPCR Master Mix (12.5 μl) (Promega, USA), 2.5 μl primers and 1.0 μl of cDNA in a total volume of 25 μl. VEGF and HIF-1α QuantiTect Sybr green primers were purchased from Qiagen, Germany. GAPDH was used as internal reference control. GAPDH primers (Invitrogen, USA) sequences used in this study were as previously mentioned [29]. The PCR condition for GAPDH, VEGF and HIF-1α comprised of first incubation at 95°C for 15 min, 40 cycles of denaturation at 95°C for 15 sec, annealing at 55°C for 30 sec, extension at 72°C for 30 sec. Fluorescence was recorded at the end of extension. A negative control without cDNA template was run simultaneously with every assay. To generate a standard curve, template cDNA from untreated-control MCF 7 cells was used. Quantification of gene expression was calculated by the standard curve and cycle threshold of each sample. The results of genes expression were normalized to reference gene expression and the fold exchange was determined in comparing with untreated cell control. Two replicates of this experiment were carried out, in which every gene had a duplicated reading. A melt curve analysis was done after QPCR to ensure the specificity of PCR product.

#### Determination of VEGF protein level

MCF 7 cells were seeded in a 96-well plate at a density of 1 × 10^5^cells/well and incubated overnight. Cells were cultured in a serum free medium for 2 h and then replaced with 10% FBS medium in presence of various doses of plant extracts at 100, 200, 300 μg/mL concentrations for 48 h under normoxic and hypoxic conditions. control wells were treated with DMSO Hypoxic condition were performed by incubating the cells in GasPak Pouch (Becton Dickinson, Sparks, Md., USA). Each concentration was prepared in triplicates. The negative control used was DMSO, with the same concentrations of the extracts. Media from each well was collected and stored at -20°C until tested. VEGF concentrations in the conditioned media were quantified by Quantikine Human VEGF ELISA kit (R&D Systems, Minneapolis, USA) according to the manufacturer’s protocol. MTT assay was used for correcting the amount of VEGF produced to the number of viable cells.

### Statistical analysis

Results were presented as means ± SD. The differences between groups were compared by the one way ANOVA followed by Tukey Post-hoc test and considered significant at *P* < 0.05. The statistical analysis was carried out by using SSPS edition 16.0.

## Results

### Rat aortic ring assay

**Figure 1:**
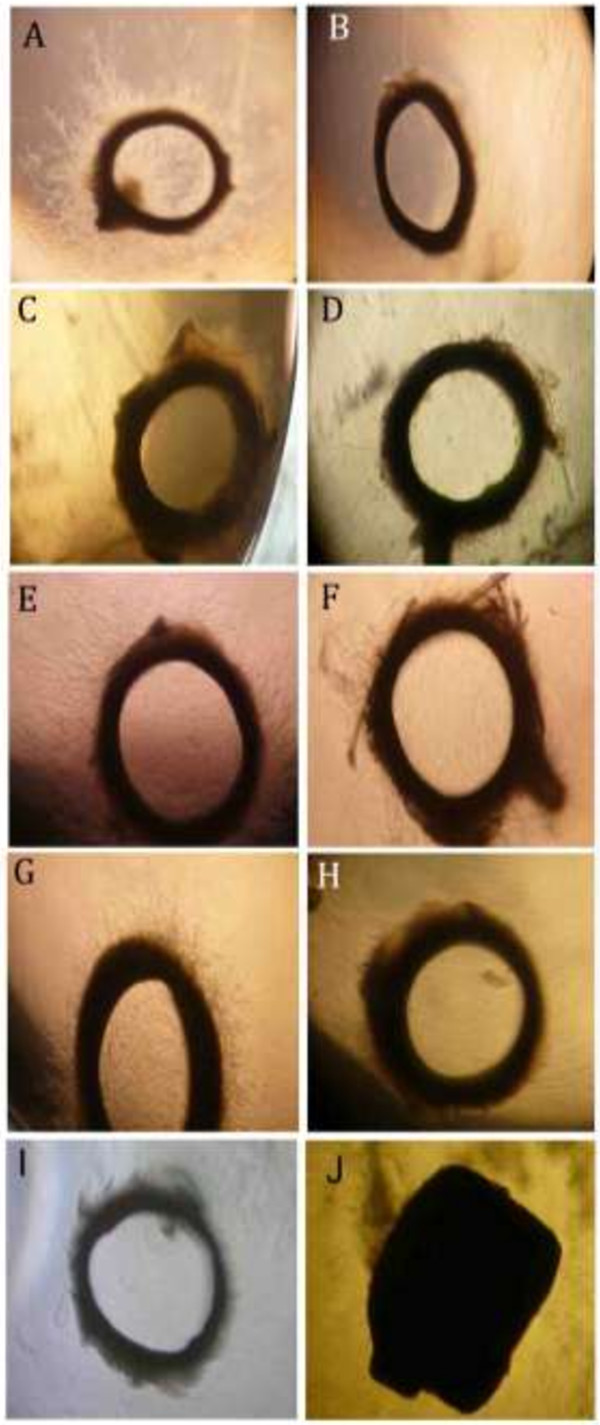
**Effect of selected plant extracts on rat aortic vessel outgrowth. (A)** negative control **(B)** positive control (Suramine) **(C, D)** 50 and 100 μg/mL of *S. dominica***(E, F)** 50 and 100 μg/mL of *S. hormium,***(G, H)** 50 and 100 μg/mL of *S. triloba* and 50 and 100 μg/mL of *S. syriaca***(I, J)**.

In order to evaluate the antiangiogenic properties of the plant extracts, we performed the rat aortic ring assay at two concentrations: 50 and 100 μg/mL (Figure 1). All of the 4 *Salvia* ethanolic extracts exhibited high inhibitory activity when tested at 100 μg/mL; they resulted in more than 70% of the mean percent inhibition to the vehicle (*P* < 0.05) (Table 1). Interestingly, at 50 μg/mL, the four extracts demonstrated different behavior. *S. dominica* and *S. syriaca* continued to show the highest inhibitory effects (Table 1 and Figure 1). While, *S. hormium* failed to keep any inhibitory activity at this concentration (*P* > 0.05) (Table 1 and Figure 1). *S. triloba* succeed in keeping its inhibitory activity and scored 77.67 ± 5.97% vessel outgrowth inhibition (*P* < 0.05) (Table 1 and Figure 1).

**Table 1:** Column 1: list of the plant extracts

**Plant name**	**Aortic assay**	**Aortic assay**	**PFL cells**
	**% of inhibition**	**% of inhibition**	**% viability**
	**(100 μg /mL)**	**(50 μg /mL)**	**(100 μg /mL)**
*S. syriaca*	100.0 ± 0.0	100.0 ± 0.0	20.2 ± 1.6
	(*P* = 0.000)	(*P* = 0.000)	(*P* = 0.000)
*S. dominica*	100.0 ± 0.0	84.1 ± 5.4	13.8 ± 7.9
	(*P* = 0.000)	(*P* = 0.000)	(*P* = 0.000)
*S. hormium*	77.1 ± 6.8	3.1 ± 1.2	111.7 ± 2.0
	(*P* = 0.000)	(*P* = 0.995)	(*P* = 0.000)
*S. triloba*	97.1 ± 0.8	77.76 ± 5.96	90.0 ± 3.7
	(*P* = 0.000)	(*P* = 0.000)	(*P* = 0.902)

Column 2 and 3: show the rat aorta ring assay results as a mean percent inhibition to the vehicle (DMSO) ± S.D at two concentrations of 50 and 100 μg/mL. Column 4: show the cytotoxic effect of the plant extracts on the proliferation of PFL cells at 100 μg/mL.

### *In vitro* cytotoxicity assay

To assess the cytotoxicity effects of the four *Salvia* extracts on normal cells, we tested the effect of those extracts at 100 μg/mL on the proliferation of periodontal ligament fibroblasts cells (PLF) in culture. As indicated in Table 1, *S. dominica* and *S. syriaca* exhibited clear cytotoxic effects by reducing the cells viability to less than 25% (*P < 0.05)*. On the other hand, *S. triloba* and *S. hormium* showed no or negligible inhibitory effect on PLF cell proliferation (*P > 0.05*).

### Antiproliferative activity against HUVECs

In order to confirm the direct antiangiogenic activity which is demonstrated in the rat aortic assay, the effects of the non-toxic extracts on PLF cells on two of HUVEC functions (proliferation and migration) were evaluated. In HUVEC proliferation assay, *S. triloba* and *S. hormium* exhibited IC_50_ of 90.0 ± 0.4 and 121.0 ± 3.5 μg/mL, respectively. The antiproliferative effect of these plant extracts on HUVECs did not result from a cytotoxic effect, because 90, 111% of the endothelial cells were alive at 50 μg/mL concentration after 72 h treatment, respectively.

### Wound healing migration assay

**Figure 2:**
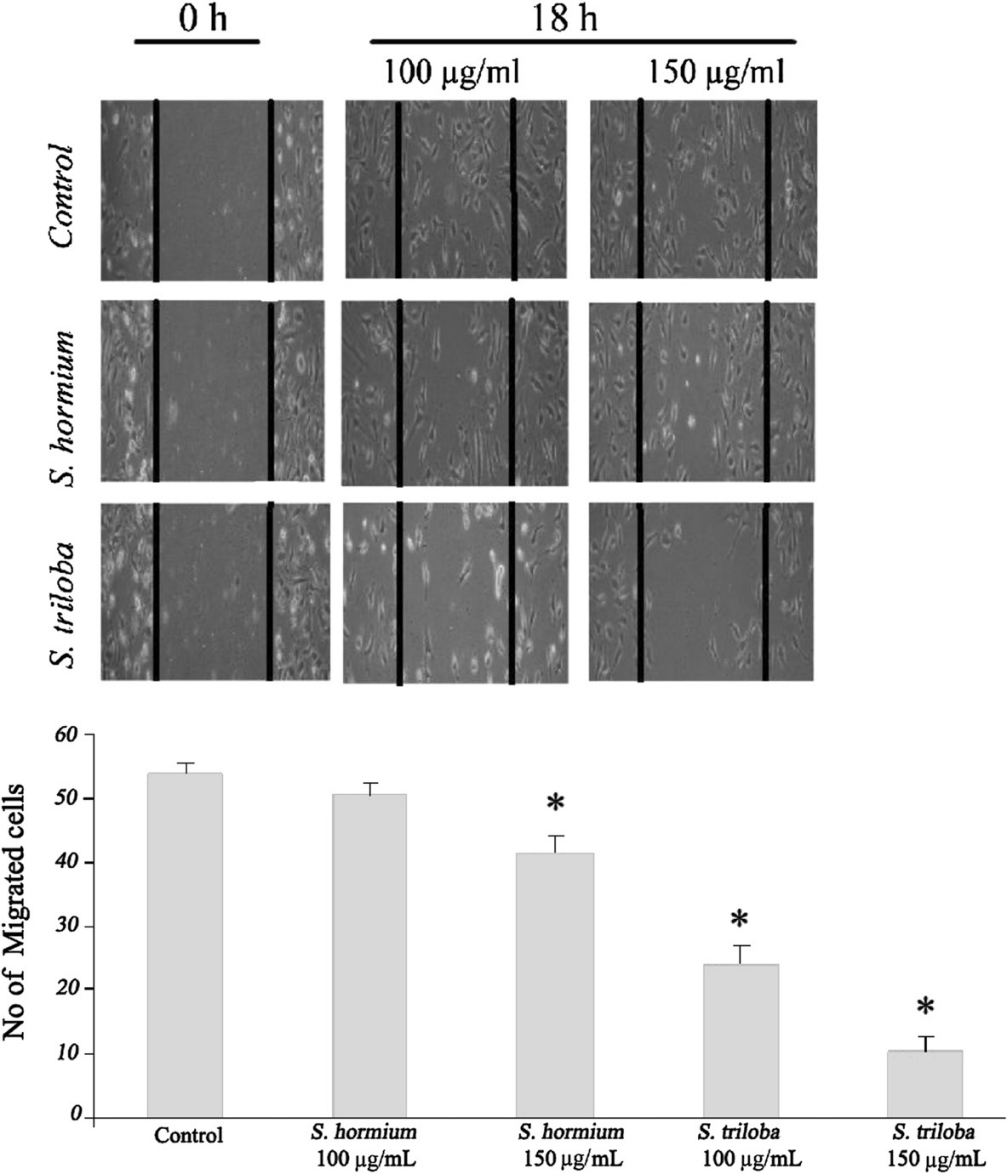
**Effects of *S. triloba *and *S. hormium *on HUVEC migration.** A scratch is created and then cells were treated with or 100 and 150 μg/mL of plant extract for 18 h. The graph represents the number of migrated cells after 18 hours treatment for the two extracts at two concentrations. *P < 0.05.

The scratch wound healing assay was performed to evaluate the effect of *S. triloba* and *S. hormium* extracts on HUVEC migration. Wound repair by endothelial cells was observed in untreated control wells. In contrast, inhibition of migration was clearly observed in wells with *S. triloba* at 100 μg/mL and 150 μg/mL concentration after 18 h incubation with 55.6 ± 5.2% *(P* = 0.000) and 80.6 ± 3.9% (*P* = 0.000) inhibition, respectively (Figure 2). While *S. hormium* at 100 μg/mL showed insignificant inhibition at 100 μg/mL with 6.48 ± 3.9% (*P* >0.05), and a modest inhibition at 150 μg/mL concentration with 23.2 ± 2.5% compared to controls (*P* =0.001) (Figure 2).

### *In vivo* CAM assay

**Figure 3:**
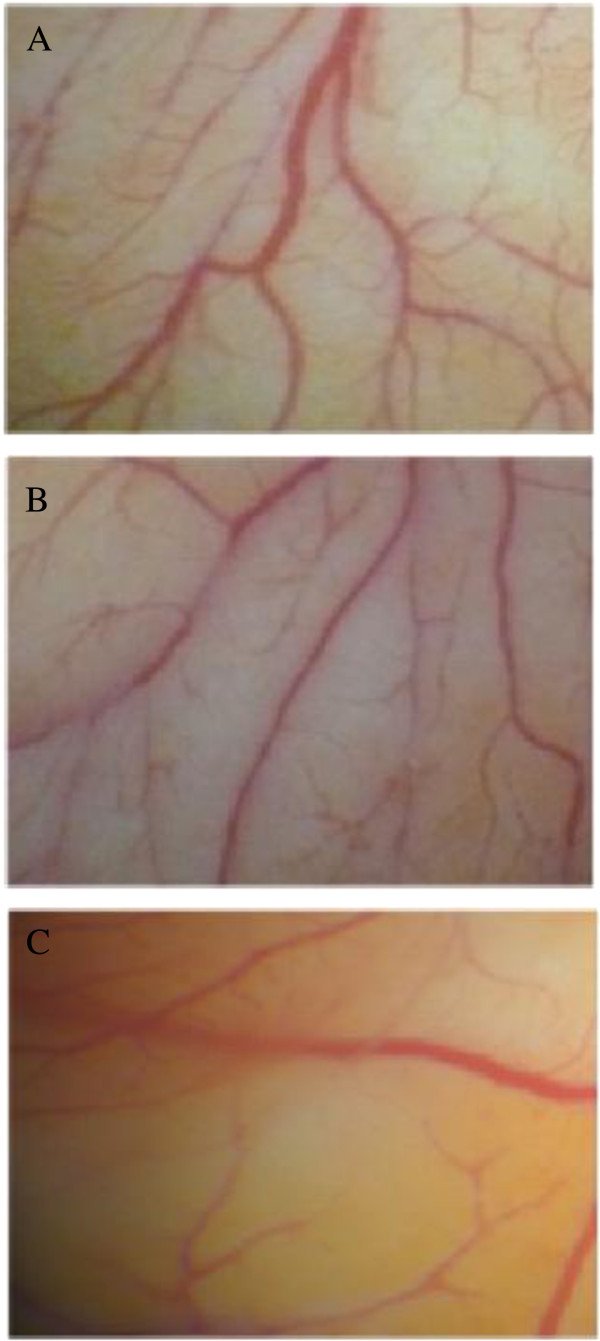
**Effect of *S. triloba *and *S. hormium *on neovascularization in CAM assay.** The embryos were treated for 24 h with **(A)** 1% DMSO as negative control, **(B)***S. hormium* 100 μg/mL and **(C)***S. triloba* 100 μg/mL.

*In vivo* antiangiogenic effect of *S. triloba* and *S. hormium* were also tested using CAM assay as an *in vivo* model at a dose of 100 μg/mL. As shown in Figure 3, a clear inhibitory activity for the *S. triloba* extract with 49.1 ± 4.1% (*P* = 0.001) inhibition was observed, while *S. hormium* extract showed insignificant inhibition 20 ± 5.3% (*P* >0.005).

### Q-PCR for VEGF and HIF-1α expression after *S. triloba* treatment

**Figure 4:**
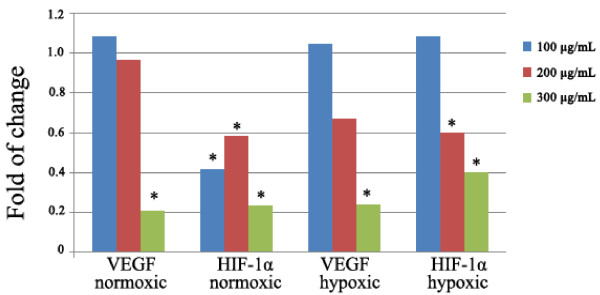
**The effect of *S. triloba *** **on VEGF and HIF-α mRNA expression after 16 h treatment at normoxic and hypoxic conditions.**

Two steps RT-QPCR were used to evaluate VEGF and HIF-1α mRNA expression in MCF 7 cells after the treatment with *S. triloba* extract. As shown in Figure 4, the *S. triloba* extract showed a significant down-regulatory effect on the expression of HIF-1α mRNA at the 3 concentrations (100 μg/mL, 200 μg/mL and 300 μg/mL) under normoxic and at 200 and 300 μg/mL under hypoxic conditions (*P* < 0.05). This inhibitory activity reached the maximum at the 300 μg/mL (*P* = 0.00), where the HIF-1α mRNA expressions were down-regulated approximately 4 and 2.5 fold at the normoxic and hypoxic conditions, respectively. On the VEGF side, the inhibition of the expression was only observed at the 300 μg/mL under both the normoxic and the hypoxic conditions (*P* < 0.05).

### Decreased levels of VEGF protein expression in MCF 7 cells after *S. triloba* treatment

**Figure 5:**
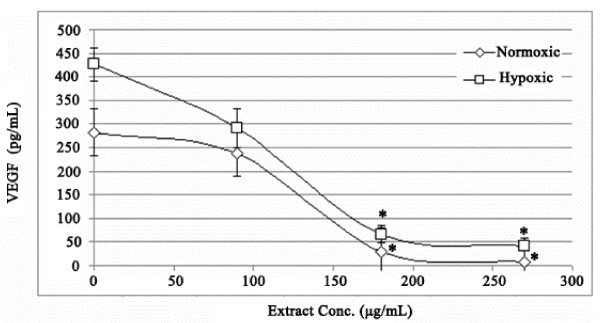
**The effect of *S. triloba *** **on VEGF protein expression after 48 h treatment at normoxic and hypoxic conditions.**

As shown in Figure 5, the *S. triloba* extract was very effective in reducing the protein level under both, hypoxic and normoxic conditions in a dose dependent manner. Interestingly, the *S. triloba* extract decreased the VEGF protein level using 1, 2, 3 times IC_50_ concentration (90, 180, 270 μg/mL) by 15.4% (*P* =0.266), 89.3% (*P* =0.000) and 97.4% (*P* =0.000) under normoxic conditions and by 31.9% (*P* = 0.000) 84.5% (*P* =0.000) and 90.2% (*P* =0.000) of inhibition under hypoxic conditions.

### Thin layer chromatography (TLC) of the extracts

All four plants showed the presence of terpenoids and flavonoids. Using preparative TLC techniques and in comparison with the reference substances, the main volatile oils components identified were alpha terpineol and 1,8- cineol and the major flavonoid traced was quercetin.

## Discussion

Angiogenesis is an essential step in solid tumour development, invasion, and metastasis. The antiangiogenesis strategy has been postulated for prevention and treatment of breast cancers [30]. There are two accepted ways to modulate angiogenesis, namely direct and indirect pathways. The direct way depends on modulating the vascular endothelial cells ability to proliferate, migrate and respond to angiogenic proteins such as VEGF. The indirect way is based on the ability to alter the expression as well as to change the activity of angiogenic proteins that activate angiogenesis. This also includes regulating the expression of the receptors on endothelial cells [31]. In the present study, the angiogenic activity of the crude ethanol extracts of four *Salvia* species grown in Jordan (*S. dominica, S. syriaca, S. triloba* and *S. hormium*) has been investigated.

Starting from the direct angiogenic findings, all four extracts have significantly inhibited the formation of new blood vessels in the rat aortic assay at 100 μg/mL. Interestingly, the reduction in the concentration to 50 μg/mL for the four extracts revealed different behavior. *S. dominica, S. syriaca, S. triloba* extracts continued to show a significant inhibitory activity (*P* < 0.05), while *S. hormium* failed to show any inhibitory activity (*P >* 0.05). To test whether this activity is a selective antiangiogenic or a result of a direct cytotoxic activity, the anti-PLF proliferative test at 100 μg/mL was performed. The results indicated that *S. dominica* and *S. syriaca* have significant cytotoxic effects (*P* = 0.000). These observations clearly point out that the strong inhibitory activity of *S. dominica* and *S. syriaca* belongs to their strong cytotoxic effects rather than their selective antiangiogenic actions. Based on these findings, these two extracts were excluded from further experiments and emphasis was given on the non-toxic extracts, *S. triloba* and *S. hormium*.

The direct antiangiogenic activity of the two extracts of *S. triloba* and *S. hormium* were tested further on two HUVEC functions, namely: endothelial cell proliferation and endothelial cell migration. These two assays represent two major steps in angiogenesis process. The anti-HUVEC proliferation results showed that the IC_50_ value of *S. triloba* and *S. hormium* are 90 ± 0.36 and 121 ± 3.47 μg/mL, respectively. The obtained IC_50_ indicated that the two extracts do possess a direct anti-proliferation activity against the HUVECs. The values also point out that the two extracts do not have a direct cytotoxic activity against HUVECs as the two IC_50_ values are far from the 20 μg/mL; the IC_50_ that has been chosen as an indication of a direct toxic activity of the plant extracts [32]. Interestingly, the results of migration assay of both extracts illustrated that *S. triloba* inhibits significantly HUVEC migration in concentration-dependent manner, while *S. hormium* showed significant inhibitory activity only at the highest concentrations (*P* = 0.000). The final step in the direct angiogenesis investigation was to test for antiangiogenic activity *in vivo*. The vascularization in chick embryo was chosen as an *in vivo* model. Again, the highly significant ability of inhibiting the formation of new blood vessels by *S. triloba* were obvious (*P* = 0.001), the finding that enforced the *in vitro* observation and voted for the high potential of *S. triloba* as inhibitor of many crucial steps of the angiogenesis process.

As to the indirect angiogenic activity, the investigation was limited to *S. triloba,* where we assessed the effect of ethanolic extracts of *S. triloba* on VEGF and HIF-1α mRNA and VEGF protein expression under normoxic and hypoxic conditions. The protein expression measurement was limited on the VEGF because it is the principle mediator of tumour angiogenesis [33]. The mRNA and protein expression measurements were conducted at two conditions, the normoxic and hypoxic conditions. The hypoxic environment was adapted to resemble the *in vivo* tumour conditions, in which the VEGF expression is known to be elevated [34,35]. Interestingly, *S. triloba* demonstrated a significant inhibitory activity on expression of the VEGF and HIF-1 α mRNA, and also on the protein expression of VEGF under normoxic and hypoxic conditions (*P* < 0.05). Interestingly, in comparison with normoxic condition, the hypoxic condition up-regulate the VEGF protein expression 1.5 times, and *S. triloba* illustrated a high potency in reversing this over-expression. Combining these indirect angiogenic findings with the fact that VEGF is one of the major HIF-1 targeted genes, specifically in recruiting the endothelial cells into hypoxic and vascular areas does point that the alteration in expression the of VEGF mRNA and protein *by S. triloba* may resulted from modulating the HIF-1 expression [10].

Taken the direct and indirect antiangiogenic investigations on crude ethanol extracts of four *Salvia* species, *S. triloba* can be nominated as a potent antiangiogenic plant that may have chemotherapeutic and/or chemoprevention potentials. Chemoprevention potentials are summarized by its direct antiangiogenic ability via the inhibition of the endothelial cell proliferation and its indirect antiangiogenic ability through inhibiting the VEGF expression. Since VEGF ligand may affect tumour vasculature in early tumour development through the recruitment of bone-marrow–derived progenitor cells that form the building blocks of a new vascular network [35], it is tempting to speculate that the antiangiogenic mechanisms described here might contribute to its angiopreventive effect. VEGF also work throughout tumour development, in which it helps existing vasculature survive, hence permitting tumours to sustain their requirements over their entire life cycle. Furthermore, the chemotherapeutic potential of *S. triloba* was noticed through significant inhibitory activity of this plant against HIF-1α mRNA expression, which is a master transcription factor related to cell proliferation/survival and resistance to chemotherapy and radiation [34]. Importantly, HIF-1α antisense therapy demonstrated a synergistic anti-tumour effect with immunotherapy [36]. Moreover, the angioprevention activity can also be speculated building on the significant inhibitory activity of *S. triloba* against HIF-1α mRNA expression. HIF-1α is one of the most important transcription factors that operate to sense environmental clues that drives angiogenesis [13]. Importantly, Hao et al. (2011) showed that RNAi targeting HIF-1α is effectively inhibiting the progression of oral squamous cell carcinoma and concluded that it may be used as a potent and specific therapy for oral cancer, especially in inhibiting and preventing cancer cell angiogenesis and survival [37].

On the other hand, the chemoprevention values perhaps will be more convincing by knowing that this plant is very popular in the Middle East, especially in the Arabic countries such as Jordan and Palestine [38,39], where people consume *S. triloba* on daily basis, either by drinking it alone or using it as a flavoring agent to black tea. Further studies are needed to include *S. triloba* to the list of dietary phytochemicals for which chemopreventive activities by interfering with multiple signaling pathways aberrant in cancer have been demonstrated [40-43].

## Conclusion

Out of the four crude ethanol extracts of four *Salvia* species grown in Jordan (*S. dominica*, *S. syriaca*, *S. triloba* and *S. hormium*). *S. triloba* has shown a potent direct and indirect antiangiogenic activity that may have chemotherapeutic and/or chemoprevention potentials. The direct antiangiogenic activity were proven using the rat aortic assay, The anti-HUVEC proliferation, migration assay and CAM assay. The indirect antiangiogenic activity were proven through assessing the effect on VEGF and HIF-1α mRNA and VEGF protein expression under normoxic and hypoxic conditions.

## Competing interests

The authors declare that they have no competing interests.

## Authors’ contribution

MZ, FA, RAD and AMSAM contributed to the design of the study, the analysis of the data, and drafted the manuscript. HS, MMS, ZDN and RN executed the experiments. All the authors have read and approved the final manuscript.

## Pre-publication history

The pre-publication history for this paper can be accessed here:

http://www.biomedcentral.com/1472-6882/13/358/prepub

## References

[B1] Folkman J Role of angiogenesis in tumor growth and metastasis Semin Oncol 2002 13 6, Supplement 16 15 18 10.1016/S0093-7754(02)70065-1 12516034

[B2] Folkman J What is the evidence that tumors are angiogenesis dependent? J Natl Cancer Inst 1990 13 1 4 7 10.1093/jnci/82.1.4 1688381

[B3] Jemal A Siegel R Ward E Hao Y Xu J Thun MJ Cancer Statistics, 2009 CA Cancer J Clin 2009 13 4 225 249 10.3322/caac.20006 19474385

[B4] Steeg PS Tumor metastasis: mechanistic insights and clinical challenges Nat Med 2006 13 8 895 904 10.1038/nm1469 16892035

[B5] Weidner N Semple JP Welch WR Folkman J Tumor angiogenesis and metastasis–correlation in invasive breast carcinoma N Engl J Med 1991 13 1 1 8 10.1056/NEJM199101033240101 1701519

[B6] Geho DH Bandle RW Clair T Liotta LA Physiological mechanisms of tumor-cell invasion and migration Physiology 2005 13 3 194 200 10.1152/physiol.00009.2005 15888576

[B7] Weigelt B Peterse JL van't Veer LJ Breast cancer metastasis: markers and models Nat Rev Cancer 2005 13 8 591 602 10.1038/nrc1670 16056258

[B8] Quesada AR Muñoz Chápuli R Medina MA Anti angiogenic drugs: from bench to clinical trials Med Res Rev 2006 13 4 483 530 10.1002/med.20059 16652370

[B9] Browder T Folkman J Pirie-Shepherd S The hemostatic system as a regulator of angiogenesis J Biol Chem 2000 13 3 1521 10.1074/jbc.275.3.1521 10636838

[B10] Pugh CW Ratcliffe PJ Regulation of angiogenesis by hypoxia: role of the HIF system Nat Med 2003 13 6 677 684 10.1038/nm0603-677 12778166

[B11] Carmeliet P Jain RK Angiogenesis in cancer and other diseases Nature 2000 13 6801 249 257 10.1038/35025220 11001068

[B12] Denekamp J Angiogenesis, neovascular proliferation and vascular pathophysiology as targets for cancer therapy Br J Radiol 1993 13 783 181 10.1259/0007-1285-66-783-181 7682469

[B13] Albini A Noonan DM Ferrari N Molecular Pathways for Cancer Angioprevention Clin Cancer Res 2007 13 15 4320 4325 10.1158/1078-0432.CCR-07-0069 17671111

[B14] Goren AC Kilic T Dirmenci T Bilsel G Chemotaxonomic evaluation of Turkish species of Salvia: fatty acid compositions of seed oils Biochem Syst Ecol 2006 13 2 160 164 10.1016/j.bse.2005.09.002

[B15] Al-Eisawi DM List of Jordan vascular plants Amman 1982 13 79 182

[B16] Lima CF Valentao PCR Andrade PB Seabra RM Fernandes-Ferreira M Pereira-Wilson C Water and methanolic extracts of Salvia officinalis protect HepG2 cells from t-BHP induced oxidative damage Chem Biol Interact 2007 13 2 107 115 10.1016/j.cbi.2007.01.020 17349617

[B17] González AG Abad T Jiménez IA Ravelo AG Luis Zahira Aguiar JG San Andrés L Plasencia M Herrera JR Moujir L A first study of antibacterial activity of diterpenes isolated from some Salvia species (Lamiaceae) Biochem Syst Ecol 1989 13 4 293 296 10.1016/0305-1978(89)90005-7

[B18] Hosseinzadeh H Haddadkhodaparast MH Arash AR Antinociceptive, antiinflammatory and acute toxicity effects of Salvia leriifolia Benth. Seed extract in mice and rats Phytother Res 2003 13 4 422 425 10.1002/ptr.1154 12722156

[B19] Hernandez-Perez M Rabanal RM de la Torre MC Rodriguez B Analgesic, anti-inflammatory, antipyretic and haematological effects of aethiopinone, an o-naphthoquinone diterpenoid from Salvia aethiopis roots and two hemisynthetic derivatives Planta Med 1995 13 6 505 509 10.1055/s-2006-959358 8824942

[B20] Alarcon Aguilar F Roman Ramos R Flores Saenz J Aguirre Garcia F Investigation on the hypoglycaemic effects of extracts of four Mexican medicinal plants in normal and Alloxan diabetic mice Phytother Res 2002 13 4 383 386 10.1002/ptr.914 12112298

[B21] Liu J Shen HM Ong CN Salvia miltiorrhiza inhibits cell growth and induces apoptosis in human hepatoma HepG2 cells Cancer Lett 2000 13 1–2 85 93 10779635 10.1016/s0304-3835(00)00391-8

[B22] Wu MH Tsai WJ Don MJ Chen YC Chen IS Kuo YC Tanshinlactone A from Salvia miltiorrhiza modulates interleukin-2 and interferon-gamma gene expression J Ethnopharmacol 2007 13 2 210 217 10.1016/j.jep.2007.05.026 17616290

[B23] Keshavarz M Mostafaie A Mansouri K Bidmeshkipour A Motlagh HRM Parvaneh S In vitro and ex vivo antiangiogenic activity of salvia officinalis Phytother Res 2010 13 10 1526 1531 10.1002/ptr.3168 20878705

[B24] Brown KJ Maynes SF Bezos A Maguire DJ Ford MD Parish CR A novel in vitro assay for human angiogenesis Lab Invest 1996 13 4 539 555 8874385

[B25] Nassar ZD Aisha AF Ahamed MB Ismail Z Abu-Salah KM Alrokayan SA Abdul Majid AM Antiangiogenic properties of Koetjapic acid, a natural triterpene isolated from Sandoricum koetjaoe Merr Cancer Cell Int 2011 13 1 12 10.1186/1475-2867-11-12 21524294 PMC3111336

[B26] Nicosia RF Lin YJ Hazelton D Qian X Endogenous regulation of angiogenesis in the rat aorta model Role of vascular endothelial growth factor Am J Pathol 1997 13 5 1379 1386 9358764 PMC1858079

[B27] Liang CC Park AY Guan JL In vitro scratch assay: a convenient and inexpensive method for analysis of cell migration in vitro Nat Protoc 2007 13 2 329 333 10.1038/nprot.2007.30 17406593

[B28] West DC Burbridge MF Three-dimensional in vitro anglogenesis in the rat aortic ring model Methods Mol Biol 2009 13 189 210 10.1007/978-1-59745-241-0_11 19301672

[B29] Kaneda R Toyota M Yamashita Y Koinuma K Choi YL Ota J Kisanuki H Ishikawa M Takada S Shimada K High‒throughput screening of genome fragments bound to differentially acetylated histones Genes Cells 2004 13 12 1167 1174 10.1111/j.1365-2443.2004.00804.x 15569149

[B30] Sogno I Venč R Ferrari N De Censi A Imperatori A Noonan DM Tosetti F Albini A Angioprevention with fenretinide: targeting angiogenesis in prevention and therapeutic strategies Crit Rev Oncol Hematol 2010 13 1 2 14 10.1016/j.critrevonc.2009.10.007 20034809

[B31] Kerbel R Folkman J Clinical translation of angiogenesis inhibitors Nat Rev Cancer 2002 13 10 727 739 10.1038/nrc905 12360276

[B32] Boik J Natural compounds in cancer therapy 2001 LLC, Minnesota, USA: Oregon Medical Press

[B33] Hicklin DJ Ellis LM Role of the vascular endothelial growth factor pathway in tumor growth and angiogenesis J Clin Oncol 2005 13 5 1011 15585754 10.1200/JCO.2005.06.081

[B34] Brown JM Giaccia AJ The unique physiology of solid tumors: opportunities (and problems) for cancer therapy Cancer Res 1998 13 7 1408 9537241

[B35] Vaupel P Kelleher DK Höckel M Oxygenation status of malignant tumors: pathogenesis of hypoxia and significance for tumor therapy Semin Oncol 2001 13 2 Suppl 8 29 35 11395850 10.1016/s0093-7754(01)90210-6

[B36] Sun X Kanwar JR Leung E Vale M Krissansen GW Regression of solid tumors by engineered overexpression of von Hippel-Lindau tumor suppressor protein and antisense hypoxia-inducible factor-1alpha Gene Ther 2003 13 25 2081 2089 10.1038/sj.gt.3302118 14595381

[B37] Zhou H Fei W Bai Y Zhu S Luo E Chen K Hu J RNA interference-mediated downregulation of hypoxia-inducible factor-1[alpha] inhibits angiogenesis and survival of oral squamous cell carcinoma in vitro and in vivo Eur J Cancer Prev 2011 13 Publish Ahead of Print: 10.1097/CEJ.1090b1013e32834dbbda 10.1097/CEJ.0b013e32834dbbda 22113109

[B38] Ali-Shtayeh MS Yaniv Z Mahajna J Ethnobotanical survey in the Palestinian area: a classification of the healing potential of medicinal plants J Ethnopharmacol 2000 13 1–2 221 232 11025160 10.1016/s0378-8741(00)00316-0

[B39] Abu-Irmaileh BE Afifi FU Herbal medicine in Jordan with special emphasis on commonly used herbs J Ethnopharmacol 2003 13 2–3 193 197 14611882 10.1016/s0378-8741(03)00283-6

[B40] Sogno I Vannini N Lorusso G Cammarota R Noonan DM Generoso L Sporn MB Albini A Anti-angiogenic activity of a novel class of chemopreventive compounds: oleanic acid terpenoids Recent Results Cancer Res 2009 13 209 212 10.1007/978-3-540-69297-3_19 19213570

[B41] Pfeffer U Ferrari N Morini M Benelli R Noonan D Albini A Antiangiogenic activity of chemopreventive drugs Int J Biol Markers 2002 13 1 70 74 10.1177/172460080301800113 12699068

[B42] Lamy E Garcia-Käufer M Prinzhorn J Mersch-Sundermann V Antigenotoxic action of isothiocyanate-containing mustard as determined by two cancer biomarkers in a human intervention trial Eur J Cancer Prev 2012 13 4 400 406 10.1097/CEJ.0b013e32834ef140 22157087

[B43] Lorusso G Vannini N Sogno I Generoso L Garbisa S Noonan DM Albini A Mechanisms of Hyperforin as an anti-angiogenic angioprevention agent Eur J Cancer 2009 13 8 1474 1484 10.1016/j.ejca.2009.01.014 19223175

