# Correction to: The antiangiogenic activities of ethanolic crude extracts of four *Salvia* species

**DOI:** 10.1186/s12906-018-2126-8

**Published:** 2018-02-16

**Authors:** Malek Zihlif, Fatma Afifi, Rana Abu-Dahab, Amin Malik Shah Abdul Majid, Hamza Somrain, Mohanad M. Saleh, Zeyad D. Nassar, Randa Naffa

**Affiliations:** 1 Department of Pharmacology, Faculty of Medicine, The University of Jordan, Amman, 11942 Jordan; 2 Department of Pharmaceutical Sciences, Faculty of Pharmacy, The University of Jordan, Amman, Jordan; 3 Department of Pharmacology, School of Pharmaceutical Sciences, Universiti Sains Malaysia, 11800 Minden, Penang Malaysia; 4 University of Queensland, School of Pharmacy, 20 Cornwall Street, Woolloongabba, QLD 4102 Australia; 5 Department of Physiology and Biochemistry, Faculty of Medicine, The University of Jordan, Amman, 11942 Jordan

## Correction

After the publication [1] it came to the attention of the authors that one of the co-authors was incorrectly included as Hamza Somrain. The correct spelling is as follows: Hamzeh Sumrein.
